# Hydrothermal Synthesis of Hematite Nanoparticles Decorated on Carbon Mesospheres and Their Synergetic Action on the Thermal Decomposition of Nitrocellulose

**DOI:** 10.3390/nano10050968

**Published:** 2020-05-18

**Authors:** Abdenacer Benhammada, Djalal Trache, Mohamed Kesraoui, Salim Chelouche

**Affiliations:** 1 UER Procédés Energétiques, Ecole Militaire Polytechnique, BP 17, Bordj El-Bahri, Algiers 16046, Algeria; nbenhammada@yahoo.fr (A.B.); kesraoui.mohamed@gmail.com (M.K.); salim.chelouche@gmail.com (S.C.); 2 Ecole Nationale Préparatoire Aux Etudes d’Ingénieur Badji Mokhtar, ENPEI, BP 5, Rouiba, Algiers 16013, Algeria

**Keywords:** carbon mesosphere, Fe_2_O_3_, supported nanoparticles, nitrocellulose, thermal decomposition, kinetics

## Abstract

In this study, carbon mesospheres (CMS) and iron oxide nanoparticles decorated on carbon mesospheres (Fe_2_O_3_-CMS) were effectively synthesized by a direct and simple hydrothermal approach. α-Fe_2_O_3_ nanoparticles have been successfully dispersed in situ on a CMS surface. The nanoparticles obtained have been characterized by employing different analytical techniques encompassing Fourier transform infrared (FTIR) spectroscopy, Raman spectroscopy, X-ray diffraction (XRD) and scanning electron microscopy (SEM). The produced carbon mesospheres, mostly spherical in shape, exhibited an average size of 334.5 nm, whereas that of Fe_2_O_3_ supported on CMS is at around 80 nm. The catalytic effect of the nanocatalyst on the thermal behavior of cellulose nitrate (NC) was investigated by utilizing differential scanning calorimetry (DSC). The determination of kinetic parameters has been carried out using four isoconversional kinetic methods based on DSC data obtained at various heating rates. It is demonstrated that Fe_2_O_3_-CMS have a minor influence on the decomposition temperature of NC, while a noticeable diminution of the activation energy is acquired. In contrast, pure CMS have a slight stabilizing effect with an increase of apparent activation energy. Furthermore, the decomposition reaction mechanism of NC is affected by the introduction of the nano-catalyst. Lastly, we can infer that Fe_2_O_3_-CMS may be securely employed as an effective catalyst for the thermal decomposition of NC.

## 1. Introduction

Cellulose nitrate, known as nitrocellulose (NC), is one of a main components of gun powder and solid propellants [1,2,3]. It has been widely investigated owing its thermal decomposition features, such as the decomposition temperature, activation energy, and reaction decomposition mechanism, notably influence the combustion behavior and/or performance characteristics of NC-based formulations [4,5]. It is recognized that the burning efficacy of solid propellants is closely dependent in the decomposition behavior of NC as well. Hence, tailoring the thermal decomposition of NC allows tuning the combustion properties of propellants containing NC. On the other hand, NC-based formulations can exhibit a low thermal stability because of the rupture of O–NO_2_ of NC even at ordinary conditions, which can cause the deterioration of their prominent characteristics and subsequently restrain their performance and safe and reliable service-life [6,7]. This situation can be prevented by incorporating either stabilizers or other additives. Thus, the investigation of the safety characteristics, namely the thermal stability, of energetic materials such as NC are indispensable for practical applications.

A few years ago, it was revealed that the incorporation of nanomaterials to NC is an efficient approach to enhance its thermal decomposition by tailoring the decomposition process and/or the activation energy without altering the safety and thermal compatibility [8,9]. Such nanomaterials may comprise metal oxides, metal nanoparticles (NPs), organometallic compounds, metallic composites, energetic nano-catalysts, and carbon nanomaterials [10,11,12]. Various metal oxide NPs have been tested as NC additives. The effect of CuO [13], Fe_2_O_3_ [14,15] nanoboron [16], bismuth oxide [17] on the thermal decomposition of nitrocellulose has been assessed and prominent results have been reported.

Hematite (α-Fe_2_O_3_) nanoparticles, one of the most stable phases of iron oxide which is an n-type semiconductor [18], have received a particular attention owing to their interesting properties and their wide range of application fields in various industrial reactions such as catalysis and biotechnology [19], lithium-ion batteries [20], gas sensing, magnetic memory, biological uses and degradation of organic contaminants [18]. α-Fe_2_O_3_ nanoparticles can be synthesized with various shapes like nano-platelets, nano-belts, nano-rods, nano-cubes, and nanotubes utilizing miscellaneous physicochemical methodologies [20,21,22,23,24]. For energetic materials applications, α-Fe_2_O_3_ nanoparticles have been used to increase the ammonium perchlorate thermal decomposition [21,25]. Zhao et al. have confirmed that Fe_2_O_3_ nanoparticles may be used safely with NC without affecting the kinetic thermal decomposition model [14,15]. Nevertheless, Fe_2_O_3_ reduces the activation energy and critical temperature of thermal explosion of NC and has a good catalytic effect by promoting the O-NO_2_ bond cleavage.

However, the catalytic performance of metal oxide nanoparticles such as Fe_2_O_3_ depends closely not only on the particles’ size and shape, but also on their distribution and dispersion. Particles with nanoscale size range are prone to aggregate because of the importance of surface energy, which will generate lowed available surface areas and reduce the catalytic efficiencies [26]. Therefore, a good dispersion of these nanomaterials using catalytic supports has drawn more attention from the scientific community. Such an efficient approach may reduce the self-aggregation drawbacks of nanoparticles and allows exploring and fully benefiting from the unique physicochemical properties of nanoparticles compared to bulk materials. To produce well-dispersed nanoparticles, numerous substances have been employed to resolve the intractable issues. Recently, carbon-based catalytic supports with outstanding features such as large surface area, chemical stability and tailorable electrical and thermal conductivity, have been revealed as useful supporting material for metal and metal oxides nanoparticles [27]. It was revealed that various metal oxide nanoparticles attached on carbon-based supports not only avoided aggregation but also improved catalytic, thermal, magnetic, and optoelectronic characteristics [28].

Several carbon catalytic supports have been reported such as graphene, graphene oxide, carbon nanotubes, fullerene and carbon mesospheres (CMS), and have been comprehensively employed in various fields [29,30,31,32,33]. CMS are a kind of carbon material, which have some specific characteristics owing to their spherical shape, such as excellent mechanical strength, high packing density, and large specific surface area [34]. Commonly, they can be readily produced by a hydrothermal carbonization procedure of organic materials like glucose [35]. CMS have attracted much interest as a catalytic support owing to the uniformity and homogeneity of their nanoparticles [36]. Their porous nanostructure and high specific area allow a large loading of metal oxide nanoparticles [37]. Nanoparticles supported on carbon mesospheres have been widely used in various field. For instance, CuO NPs supported on CMS have been used for sensing application and super capacitors [36,38], whereas ZnO NPs find utilization as photocatalysts [22], catalysis [39], and other applications [40].

To the best of the authors’ knowledge, a research gap still exists in investigation the effect of Fe_2_O_3_ nanoparticles supported on carbon mesospheres as a catalyst for the thermal decomposition of nitrocellulose. Thus, this work deals with the synthesis of carbon mesosphere CMS as catalytic support and α-Fe_2_O_3_ nanoparticles decorated on carbon mesospheres, respectively. Then, the catalytic effect of the synthesized materials has been evaluated using differential scanning calorimetry (DSC). The kinetic parameters, i.e., the activation energy (*E*a), the pre-exponential factor (*A*) and reaction model were computed through the isoconversional analysis using four kinetic methods, explicitly, *it*-FWO (iterative Flynn–Wall–Ozawa), *it*-KAS (iterative Kissinger–Akahira–Sunose), TAS (Trache–Abdelaziz–Siwani), and Vyazovkin’s equation.

## 2. Experiment and Methods

### 2.1. Materials

Nitrocellulose with nitrogen content of 12.56% was produced using the methodology mentioned in our recent works [2,41,42]. Different analytical chemicals comprising glucose (C_6_H_12_O_6_) for CMS preparation, iron chloride (FeCl_3_·6H_2_O), as iron precursor, and ammonia NH_4_OH as reducing agent for iron oxide synthesis, were provided by VWR chemicals (100 Matsonford Road, Radnor, PA, USA) and used without further purification. Absolute ethanol and distilled water have been used to purify the obtained catalyst. 

### 2.2. Carbon Mesospheres (CMS) and Fe_2_O_3_-CMS Preparation

CMS were prepared using a simple hydrothermal treatment of glucose as precursor as reported elsewhere [43], with slight modifications. Typically, a 0.5 M solution of glucose was kept in an autoclave introduced in an oven at 180 °C for 6 h and then cooled at room temperature. A black residue was recovered by centrifugation and washed three times with absolute ethanol and distilled water. Finally, the product obtained was dried at 60 °C in an oven for 8 h.

Fe_2_O_3_-CMS composite was prepared by a hydrothermal method as follows. First, 1 g of the prepared CMS was dispersed with vigorous stirring in 80 mL of distilled water. An iron solution was simultaneously prepared using iron chloride as precursor in distilled water (5 g, 100 mL). The two solutions were then mixed under stirring for 45 min. After that, 34% ammonia solution was dropwise added to a stirred mixture until a pH of 8. The obtained solution was then incorporated in Teflon-sealed autoclave and heated at 180 °C for 24 h. After being cooled at room temperature, the dispersion was centrifuged, washed several times using distilled water and absolute ethanol, and dried in oven at 60 °C for 8 h, and stored for further characterizations.

### 2.3. Preparation of CMS-Nitrocellulose (NC) and α-Fe_2_O_3_-CMS-NC Composites

**Figure 1:**
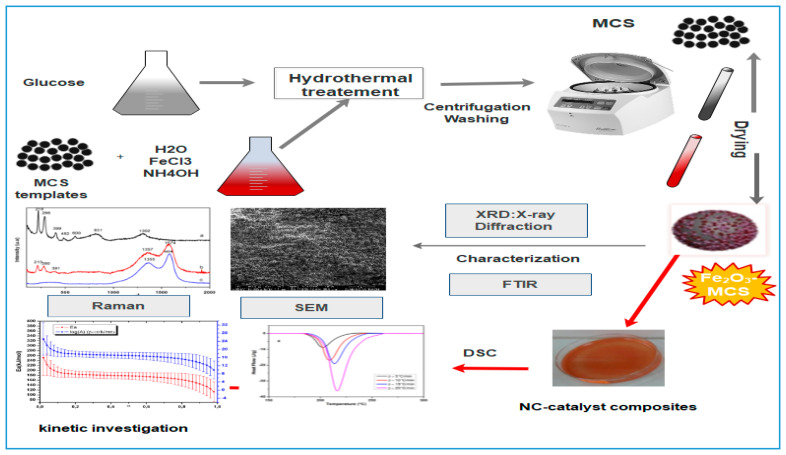
Schematic illustration of preparation and characterization of carbon mesosphere (CMS) and Fe_2_O_3_-CMS composites.

In order to obtain a good dispersion of the prepared nano-catalysts within the NC matrix, NC-catalyst films were prepared via a dissolution method. In a typical experiment, after being dried in an oven at 60 °C for 24 h, 0.5 g of nitrocellulose was dissolved in 30 mL of acetone under stirring. Then, 25 mg of catalyst, with mass ratio NC:catalyst of (95%:5%), was gradually added under stirring. After a sonication for 20 min, the obtained colloidal mixtures were consistently spread in glass Petri dishes at room temperature until the total elimination of acetone, forming thin films of NC-CMS and NC-Fe_2_O_3_-CMS, respectively. A pure NC film was also produced with similar method without the addition of catalyst. The experimental procedure was schematized in Figure 1.

### 2.4. Samples Characterization

#### 2.4.1. Raman Spectroscopy and Fourier Transform Infrared Spectroscopy Analyses

As an imperative nondestructive analytical tool to investigate the chemical composition and the structure of a variety of materials, Raman spectroscopy has been proved as an appropriate technique for the characterization of nanomaterials since it allows detecting characteristic vibrations with low intensities [44,45]. These vibrational features of the produced nanoparticles were determined by employing Raman spectroscopy (Thermo Scientific DXR, Waltham, MA, USA). The chemical functions of the prepared catalysts were investigated using Fast Fourier infrared transform (Bruker-Vertex 70, Rudolf-Plank-Str., Ettlingen, Germany) in attenuated total reflectance (ATR) mode in the wavenumber range 400–4000 cm^−1^. 

#### 2.4.2. Structural and Morphological Investigations

The phase purity of CMS and Fe_2_O_3_-CMS was assessed by using PANalytical X’Pert PRO X-ray diffractometer (XRD, Westborough, MA, USA) at 40 mA and 45 kV with Cu anode Kα radiation (λ = 1.54 Å) from 20 to 70° (2θ) at a Step Size of 0.0170. A FEG JSM 7100F TTLS scanning electron microscope (SEM) (JEOL, Leuvensesteenweg, Zaventem, Belgium) was employed to determine the morphology and the particles size of the obtained nanoparticles. The micrographs were acquired with an accelerating voltage of 2 kV. To guarantee reproducible data, more than 50 nanoparticles were used. The particle size of the catalysts was estimated by Image J software (National Institutes of Health by an employee of the Federal Government, MD, USA).

#### 2.4.3. Thermal Analysis

The influence of the incorporated nanocatalysts on the thermal decomposition of NC was assessed by a Perkin Elmer differential scanning calorimeter (DSC, Waltham, MA, USA). For each measurement, 0.8–1 mg of fine cut film is place in a closed aluminum pan. The DSC experiments were realized within the temperature range of 50–300 °C at various heating rates (5, 10, 15 and 20 °C/min) under nitrogen atmosphere (20 cm^−3^/min). Analysis uncertainties are lower than 0.2 °C for the temperature.

### 2.5. Kinetic Parameters Determination

A few years ago, the International Confederation for Thermal Analysis and Calorimetry (ICTAC) kinetics committee has claimed that the isoconversional methodology is the utmost appropriate methodology to study the kinetic of thermally stimulated reactions [46]. Based on the use of multiple heating rates rather than isothermal methods, this allows consistent kinetic triplets to be obtained, encompassing, the activation energy, the pre-exponential factor and the most suitable reaction model [47,48].

The reaction rate of solid-state thermal decomposition can be written in terms of T and α as [46,49]:

(1) $$\frac{\;d\alpha }{dt}=k\left(T\right)f\left(\alpha \right)$$

where α, *t*, *T*, *k*(*T*) and *f*(α) refer, respectively, to the extent of conversion (0 < α <1), the time, temperature, the rate constant, and the reaction mathematical function model that denotes the reaction mechanism. The values of α are experimentally determined from the DSC data as the ratio of the current physical feature change to the total change of this property in the process. Using DSC analysis, α is given as:

(2) $$\alpha \;=\frac{\int_{t0}^{t}\frac{dH}{dt}\;dt}{\int_{t0}^{t\infty }\frac{dH}{dt}\;dt}=\frac{\Delta H}{\Delta H\mathrm{tot}}$$

where $\frac{dH}{dt}$ is the heat flow, Δ*H* is the current heat change and Δ*H*_tot_ is the total heat change determined by DSC.

The substitution of *k*(*t*) by its expression from Arrhenius equation leads to:

(3) $$\frac{d\alpha }{dt}=A\exp\left(-\frac{Ea}{RT}\right)f\left(\alpha \right)$$

where *A* is the pre-exponential factor, *E*_a_ is the apparent activation energy of the decomposition reaction and *R* is the universal gas constant.

In the case of multiple heating rate programs, the introduction of heating rate *β* ($\beta =\frac{dT}{dt}$) transforms Equation (3) to:

(4) $$\frac{d\alpha }{dT}=\frac{A}{\beta }\exp\left(-\frac{Ea}{RT}\right)f\left(\alpha \right)$$

By integration, one obtains the integral form of the reaction model *g(α*), and the 41 forms are reported by Trache et al. [50]:

(5) $$g\left(\alpha \right)=\overset{\alpha }{\underset{0}{\int }}\frac{d\alpha }{f\left(\alpha \right)}=\frac{A}{\beta }\overset{T}{\underset{0}{\int }}\exp\left(-\frac{Ea}{RT}\right)\mathrm{dT}$$

As the integral of the temperature dependence part of Equation (5) does not have an analytical solution for an arbitrary temperature program, different approximate equations have been suggested in the literature in order to carry out the kinetic analysis leading to approximate integral methods such as Doyle [51], Coats–Redfern [52] and Senum and Yang [53].

In the present work, to evaluate the kinetics parameters, we have employed four isoconversional methods, i.e., *it*-KAS [54], *it*-FWO [54], TAS [50] and Vyazovkin’s equation (VYA/CE) [55]. The details of these methods were given in our previous works [42,50].

## 3. Results and Discussion

### 3.1. Characterization of CMS and Fe_2_O_3_-CMS

**Figure 2:**
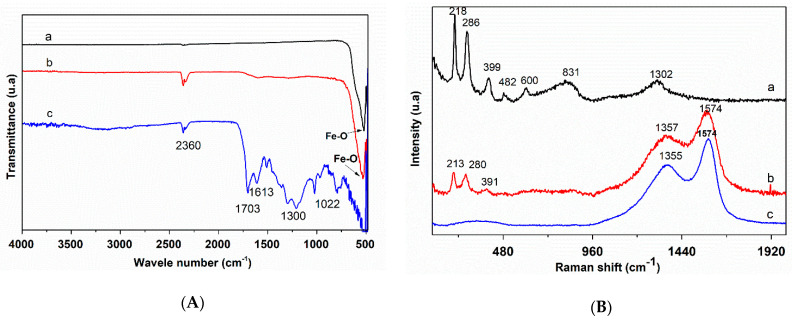
(**A**) Fourier transform infrared (FTIR) spectra of: (a) commercial hematite, (b) Fe_2_O_3_-CMS, (c) CMS, (**B**) Raman spectra of: (a) commercial hematite, (b) α-Fe_2_O_3_-CMS, (c) CMS.

Figure 2A shows the FTIR spectra of the prepared carbon mesospheres and iron oxide nanoparticles decorated on carbon mesospheres, as well as that of the commercial hematite (α-Fe_2_O_3_). For the CMS spectrum, the peak absorption at 1703 and 1613 cm^−1^ could be attributed to C=O and C=C vibrations, respectively. The broad absorption peak around 3400 cm^−1^ and the band in the range of 1000–1300 cm^−1^ are attributed to the stretching vibrational modes of C–OH bond and O–H bending [43], indicating the existence of large amount of hydroxyl groups on the surface of CMS.

After being decorated with iron oxide nanoparticles, the band at 1703 cm^−1^ has disappeared demonstrating the cleavage of C=C bonds. Furthermore, the absorption peak intensities of the band at 3400 cm^−1^ and the band in the range of 1000–1300 cm^−1^ are significantly decreased, revealing the formation of metal-oxygen (Fe–O) bonds [56,57,58]. This finding can be confirmed by the appearance of an absorption peak at around 523 cm^−1^, which is attributed to stretching vibrational modes of metal-oxygen (Fe–O) bonds. Similar absorption bands have been also found in the pure commercial hematite at 523 cm^−1^. An absorption peak at 2358 cm^−1^ for the three curves corresponded to the asymmetric stretching of the adsorbed CO_2_ during sample synthesis [23]. These results indicate the existence of α-Fe_2_O_3_ nanoparticles on the surface of carbon mesosphere as well. On the other hand, Raman spectra of the prepared catalyst as well as the commercial hematite specimen are displayed in Figure 2B. The spectra of CMS and Fe_2_O_3_-CMS displayed two characteristic bands. The first at 1357 cm^−1^, assigned to the D band, is related to structural defects, whereas the second at 1574 cm^−1^ corresponded to the G band, representing the graphitic structure in carbon materials [38,59]. It has been reported that the D/G ratio of band intensities represents the graphitic structure with respect to the structure disorder [60,61]. These structural defects are mainly due to surface groups containing oxygen [62]. In addition, the bands at 213 and 480 cm^−1^ (218 and 482 cm^−1^ for the commercial hematite) belonged to two A_1g_ symmetry species. However, the peaks at 280 and 391 cm^−1^ (286 and 399 cm^−1^ for the commercial hematite) were assigned to Eg symmetry as a characteristic Raman phonon bands for Fe_2_O_3_ These bands were observed with a slight attenuation for Fe_2_O_3_-CMS [24,63]. A red shift for Fe_2_O_3_-CMS Raman peaks, in comparison with the commercial sample, is detected which is due to the nanoparticles size reduction. Furthermore, these results indicate the production of -Fe_2_O_3_ nanoparticles on the surface of CMS.

**Figure 3:**
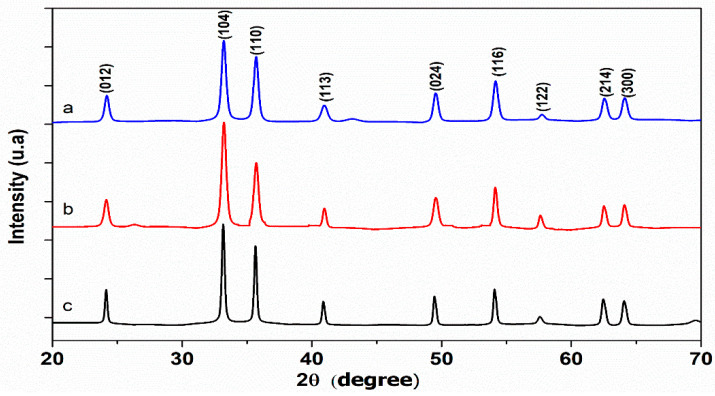
X-ray diffraction (XRD) patterns of (**a**) Fe_2_O_3_, (**b**), Fe_2_O_3_-CMS and (**c**) CMS.

Figure 3 shows the diffraction peaks of the synthesized catalyst. The XRD pattern of carbon mesospheres indicates the amorphous character of the synthesized mesospheres with one large diffraction peak at 2θ = 24° [36]. The peaks at 24.13, 33.15, 35.62, 40.87, 49.40, 53.98, 57.53, 62.41, 64.04 and 71.92°, corresponding to (012), (104), (110), (113), (024), (116), (122), (214) and (300) planes of Fe_2_O_3_, respectively, match well the rhombohedral Fe_2_O_3_ hematite with a space group R3c and unit cell parameters a = 5.038 Å and c = 13.772 Å (JCPDS Card No. 33-664) [24]. These peaks are found for both Fe_2_O_3_ nanoparticles and Fe_2_O_3_-MSC. Besides, the XRD spectra reveal that the peaks of Fe_2_O_3_ nanoparticles are more intense and sharper compared to those of Fe_2_O_3_-MSC, indicating the high crystallinity of iron oxide [64], and show a strong preferential orientation of (104) and (110) planes [23]. The average crystallite size diameter D for the prepared Fe_2_O_3_ nanoparticles, is determined from the diffraction peak widths, employing Debye–Scherrer’s equation:

(6) $$D\;=\frac{k.\lambda }{\beta \cos\theta }$$

with D: crystallite size diameter, k shape factor (k = 0.94), λ: Cu-Kα anode radiation wavelength (λ = 1.54 Å), *β*_hkl_: full width at half maxima value (FWHM) in radians, and θ the scattering angle. The computed average crystallite size diameter D is indicated to be in the range of 33 nm.

The XRD result of Fe_2_O_3_-MSC indicates also the presence of both iron oxide nanoparticles and carbon mesospheres.

**Figure 4:**
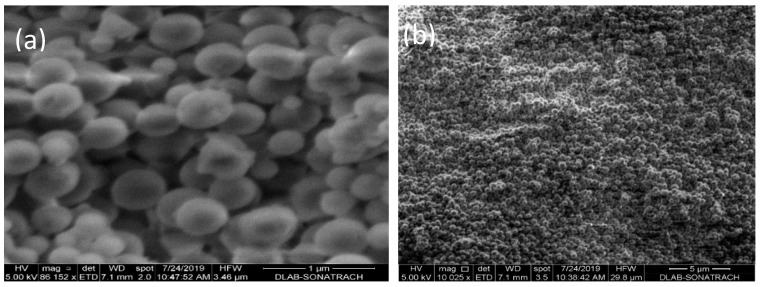

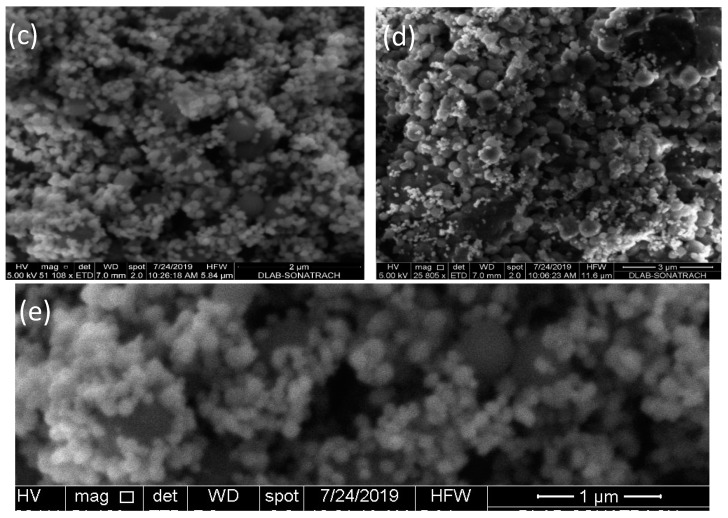
Scanning electron microscope (SEM) images of (**a**,**b**) CMS and (**c**–**e**) Fe_2_O_3_-CMS.

Figure 4 and Figure S1 show the morphology of the prepared CMS and Fe_2_O_3_-CMS. The SEM images of CMS (a and b) indicate a uniform and homogenous spherical shape with a particle size of about 334.5 nm (Figure S2 and Table S1). From micrographs c, d, and e, the Fe_2_O_3_ nanoparticles could easily be observed on the surface of carbon mesospheres with a relatively uniform distribution. The particle size of the supported Fe_2_O_3_ nanoparticles is around 80 nm (Figure S3 and Table S2). These results further indicate that the extern surface of carbon mesospheres act as a template for growing the iron oxide nanoparticles.

### 3.2. Thermal Analysis

**Figure 5:**
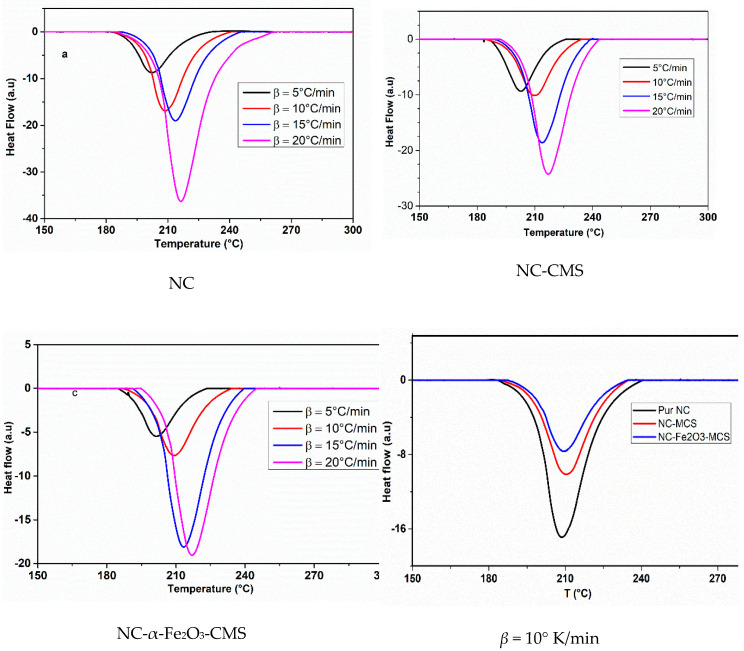
Differential scanning calorimetry (DSC) thermograms of pure nitrocellulose (NC), NC-CMS and NC-Fe_2_O_3_-CMS composites.

To evaluate different material combinations and ensure the safety during production and storage of energetic materials, compatibly is an important parameter that should be taken into account [65]. Among the various techniques used to evaluate compatibility, DSC thermal analysis is widely employed owing its outstanding features [6,7,42,66]. In order to investigate the compatibility and the effect of the prepared catalysts on the thermal decomposition of nitrocellulose, DSC analyses have been performed at different heating rate and the obtained thermograms are given in Figure 5. The three systems (Pure NC, NC-CMS, NC-Fe_2_O_3_-CMS) present the same trend, indeed, one exothermic peak is observed and corresponded to the decomposition of NC [67,68]. With the increase of the heating rate, the peak temperature shifts to higher values. Such results are in good agreement with other works [69]. According to the values of the onset and peak temperatures, given in Table 1, one can observe that the introduction of catalyst has slightly increased the peak temperature. Indeed, for *β* = 10 °C/min (Figure 5), pure NC decomposes 1.1 °C earlier than NC-CMS and 1.2 °C earlier than NC-Fe_2_O_3_-CMS. Considering the NC system with and without CMS/CMS-Fe_2_O_3_, the peak temperatures of DSC curves increase with the addition of CMS and CMS-Fe_2_O_3_, respectively. As the heating rate increases, the exothermic peak becomes sharper indicating a faster chemical reaction [70].

Moreover, From Table 1, it can be inferred that the shifts in the decomposition temperature (*β* = 5 °C/min) for NC + MCS and NC+ Fe_2_O_3_-MCS are 0.3 K and 0.5 K, respectively. These shift values are small enough to conclude that the two additives are compatible with NC, even though the STANAG 4147 [71] standard recommends DSC experiments performed on mixtures prepared in 1:1 (*w/w*) at *β* = 2 °C/min. These results indicate the high compatibility of CMS and Fe_2_O_3_-CMS with NC [72], and accordingly Fe_2_O_3_-CMS may be employed as nanocatalyst in the production of NC-based propellant formulations [14].

### 3.3. Kinetic Parameters

Exploring the DSC values obtained at various heating rates, *it*-KAS, TAS, *it*-FWO and VYA/CE have been used to investigate the thermal decomposition kinetics and thus, evaluate the kinetic parameters, i.e., the activation energy *E*_a_, the pre-exponential factor *A* and the most probable decomposition model g(α). Numeric calculations were carried out using a MATLAB interface.

**Figure 6:**
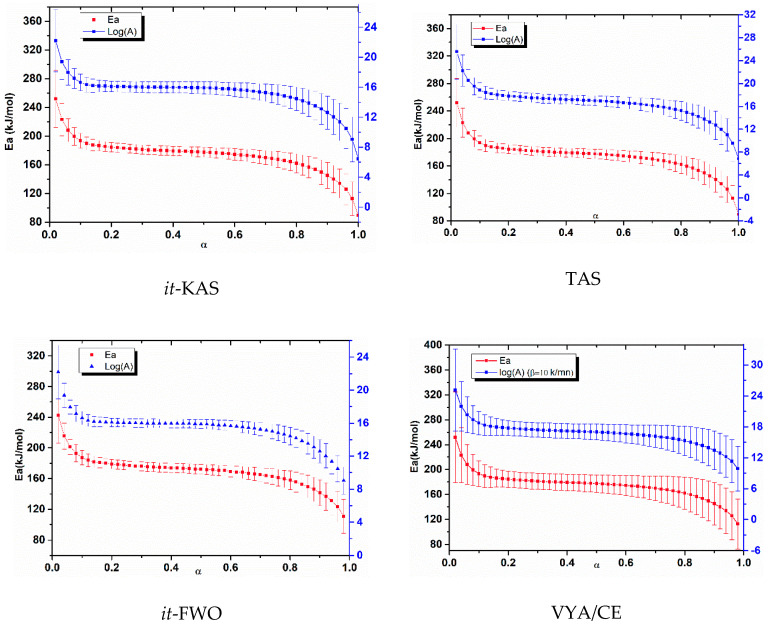
Arrhenius parameters and their associated confidence intervals evolution with respect to α determined by the different kinetic methods for pure NC.

**Figure 7:**
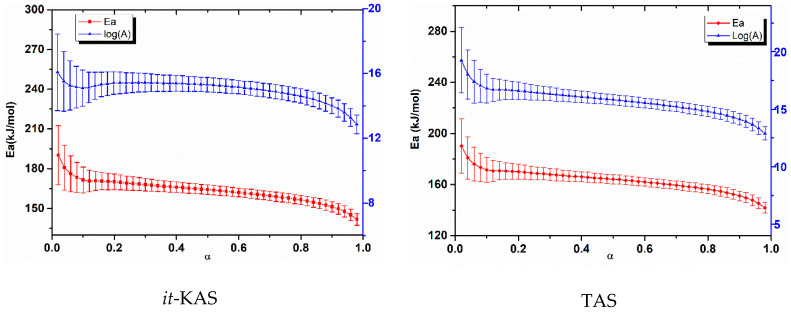

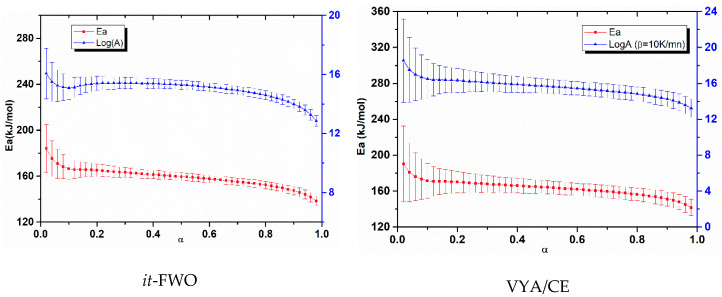
Arrhenius parameters and their associated confidence intervals evolution with respect to α determined by the different kinetic methods for NC-Fe_2_O_3_-CMS.

The kinetic parameters as well as their corresponding confidence intervals evolution determined by the different isoconversional methods for NC and NC-Fe_2_O_3_-CMS are depicted in Figure 6 and Figure 7, respectively. Likewise, the mean values of activation energy, pre-exponential factor and the most probable reaction mechanism g(α) for the three studied systems are given in Table 2.

The accuracy of the obtained activation energy and the pre-exponential factor values for *it*-KAS, TAS and *it*-FWO is confirmed with the linear correlation coefficient (R^2^), which is found to be in the range (0.95626 to 0.99993). Furthermore, the obtained values of the activation energy and pre-exponential factor allowed us to check that the used isoconversional methods provided close values of *E*_a_ and *A* with a relative deviation of 15.85% and 23.35% for NC, 8.09% and 11.54% for NC-CMS and 7.88 and 11.60% for NC-Fe_2_O_3_-CMS, respectively.

The error bars obtained for both *E*_a_ and log(*A*) are very close, indicating the accuracy of implemented computations [69]. The differences that appeared are undoubtedly caused by the different approximations used by the employed kinetic methods.

**Figure 8:**
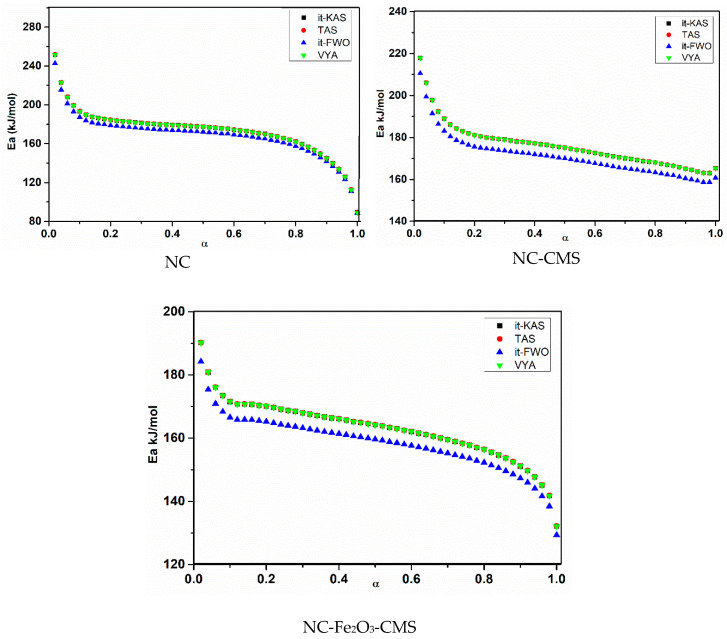
Activation energy (*E*a) evolution for the investigated systems by using different isoconversional methods.

**Figure 9:**
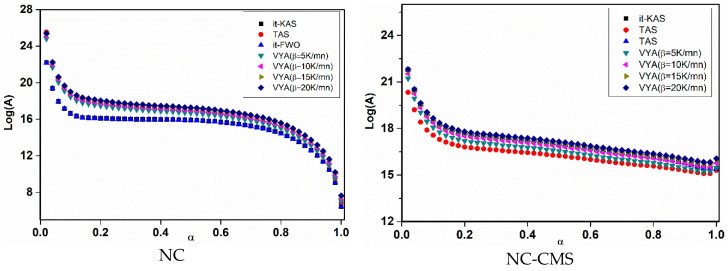

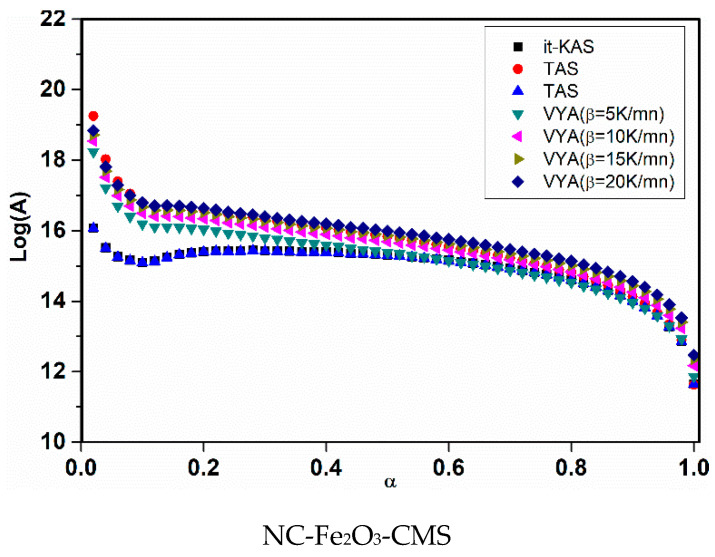
Pre-exponential factor evolution) for the investigated systems by using the different kinetic methods.

Moreover, Figure 8 and Figure 9, show respectively, the evolution of a *E*_a_ and log(*A*) against α using the four isoconversional methods for NC, NC-CMS and NC-Fe_2_O_3_-CMS. The results obtained show the same trends for *E*_a_ and log(*A*) evolution with a slight difference between models. Indeed, for the four employed isoconversional models, the values obtained seem to be close to each other with slight inferior values for *it*-FWO kinetic method. On the other hand, for the extent of conversion between 0 and 0.01, *E*_a_ and log(*A*) turn out to be more important what is attributed to the cleavage of O-NO_2_ linkages in nitrocellulose and the liberation of NO_2_ chemical groups. Then, the two parameters decrease until the end of the reaction. This behavior could be attributed to the autocatalytic parallel reactions, which can generate reactive species that may accelerate the thermolysis and hydrolysis processes [67,68]. As a very strong oxidizing agent, NO_2_ stagnates in the polymer skeleton and then reacts with the RO^•^ radicals or their degradation products, causing the opening of the NC anhydroglucopyranose rings to generate other released gases [15,73]. Moreover, the values of *E*_a_ and log(*A*) obtained are within the range of 158.278–176.137 kJ/mol for *E*_a_ and 15.2281–17.2619 (s^−1^) for log(*A*) which correspond to the common rage values of energetic materials [14].

Regarding the catalytic activity of the incorporated nanocatalyst, one can observe that the addition of carbon mesospheres, acting as catalytic support, has slightly increased the activation energy based on the four used kinetic models, and thus may have a stabilizing effect on the thermal decomposition of NC. Other carbon nanomaterials such as carbon nanotubes, nanodiamond, and graphene oxide (GO) have been revealed to play stabilizing effect on some organic energetic materials owing to their great thermal conductivity [28]. In our case, CMS facilitates the heat transfer from the reaction zone to the unburned portion of NC, which sustains the propagation of the exothermic reaction. The improvement in heat conductivity would result in less heat accumulation and low hotspots formation, which are important factors determining the sensitivity and stability of energetic materials [74]. Moreover, as shown in Table 1, CMS increases slightly the energy release of NC (∆*H* (J/g)) through the improvement of the contact between fuel/oxidizer species of NC due to the better dispersion of CMS within NC matrix. Thiruvengadathan et al. have demonstrated a similar effect for nanothermites supplemented with GO [75].

After the decoration of CMS with Fe_2_O_3_ nanoparticles, this trend has changed and the activation energy decreases by 12.9 kJ/mol, indicating the good catalytic activity of this additive on the decomposition behavior of nitrocellulose. It was reported that the presence of GO-based catalyst slightly improved the thermal stability (higher decomposition temperature) of 1,3,5,7-tetranitro-1,3,5,7-tetrazocane (HMX) and decreased the activation energy [76]. In another work, Chen et al. have evaluated the effect of GO-Ni on the thermal decomposition of triaminoguanidine nitrate and found a similar trend, where an increase of thermostability but lower energy barrier for the thermal decomposition have been mentioned [70]. On the other hand, the agglomeration problem of nano-sized catalysts is excluded in the presence of CMS as the supported dispersion media, resulting in better contact of Fe_2_O_3_ nanoparticles with NC fibers. The Fe_2_O_3_-CMS would show strong catalytic effect only in gas-phase reaction after initial decomposition of the NC at higher temperature (increased *T*_peak_), resulting in lowering activation energy of decomposition. The catalytic effect of Fe_2_O_3_-CMS on the NC themolysis reaction occurs mostly in the gas phase, where the reaction could be accelerated upon initial decomposition of the NC fibers. The presence of CMS would, however, prevent the initial decomposition of NC due to its high thermal conductivity. Similar trend has been recently reported by He et al., who investigated the thermal decomposition of the 1,3,5-trinitro-1,3,5-trizocane (RDX) supplemented with GO-based catalyst [77]. Furthermore, as displayed in Table 1, Fe_2_O_3_-CMS increases sensibly the energy release of NC through the improvement of the contact between fuel/oxidizer species of NC due to the better dispersion of Fe_2_O_3_-CMS within NC matrix.

On the other hand, the same trend of both activation energy and pre-exponential factor with respect to α is well represented by the compensation effect. Therefore, in our work, the compensation effect has been investigated using TAS and VYA/CE models for different heating rates of by plotting Log(*A*) as a function of *E*_a_ allowing us to compute the compensation parameters as displayed in Table 3. The obtained values of linear correlation coefficient (R^2^) confirm the good compensation effect between Log(*A*) and *E*_a_.

**Figure 10:**
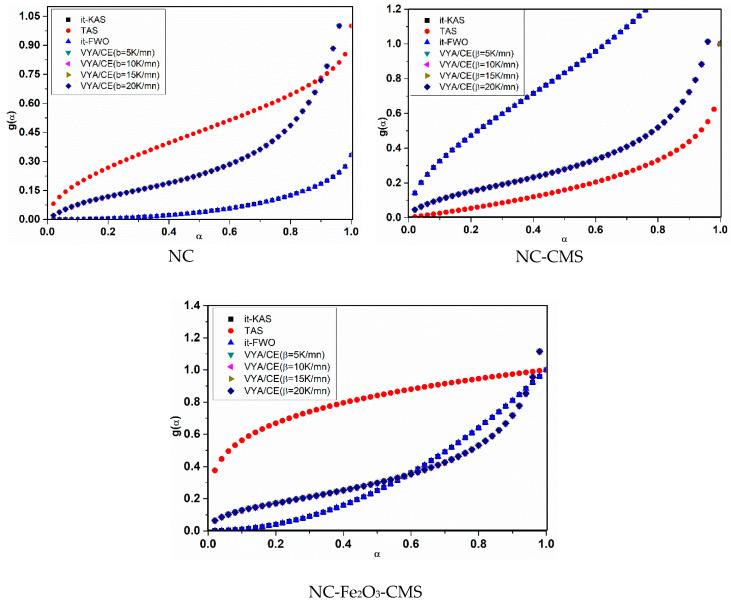
Integral reaction model evolution for the different systems studied.

Regarding the reaction model, among the 41 available, the evolution of the integral reaction mechanism values *vis* α displayed in Figure 10 and the most probable models g(α) are reported in Table 3. Various models can be attributed to NC decomposition either pure or with catalyst, which is dependent on the chosen kinetic method. NC decomposes according to G_7_ = $[1-{\left(1-\alpha \right)}^{\frac{1}{2}}{]}^{1/2}$ with the TAS model and the three-dimensional diffusion using *it*-KAS and *it*-FWO. The incorporation of carbon mesospheres could stabilize NC through the decrease of the heat accumulation and preventing the hotspots formation during the decomposition. Therefore, CMS could change the decomposition mechanism from G_7_ model to others. With *it*-KAS and *it*-FWO, NC-CMS decomposes according to a random nucleation mechanism (Avrami–Erofeev) A_2_ = $-\ln{\left(1-\alpha \right)}^{\frac{1}{2}}$ and a chemical reaction for the TAS model. Similar behavior has been reported by Sánchez-Jiménez, showing that the addition of clay nano-flakes produced a change of the thermal degradation mechanism towards a nucleation and growth model [78]. The authors indicated that such mechanism change is likely to be responsible for the increased stability.

For NC-Fe_2_O_3_-CMS, it decomposes according to a nucleation power and parabolic low (P_2_ = $\alpha^{2}$P_1/3_ = $\alpha^{\frac{1}{3}}$) for the three isoconversional models, revealing that Fe_2_O_3_ behaves as the catalytic center for which the decomposition depends considerably on the nucleation sites of Fe_2_O_3_. Similar result has been reported by Shen et al., who determined the decomposition mechanism of triaminoguanidine nitrate supplemented with GO-Ni [70].

Recently, our research group revealed that NC containing Fe_2_O_3_ nanoparticles using iron chloride as precursor decomposes following the random nucleation mechanism (Avrami–Erofeev) [4]. In other works, chemical reaction mechanism $F_{3/4}=1-{\left(1-\alpha \right)}^{\frac{1}{4}}$ has been assumed for pure NC and NC-Fe_2_O_3_ composite utilizing iron chloride as precursor [14], and for NC-Al/Fe_2_O_3_ mixture [15]. More recently, Chellouche et al. [42] attributed a nucleation (parabolic law) for nitrocellulose. Such differences in the kinetic could be assigned to the difference in the composition that influence the NC stability and its decomposition behavior [79] as well as the difference in the employed analytical tool and the kinetic methodologies. 

## 4. Conclusions

From the foregoing experiments and modeling, the following conclusions regarding preparation of the nano-catalyst as well as its catalytic effect on NC decomposition may be drawn:

(1) Carbon mesospheres CMS and iron oxide nanoparticles decorated on carbon mesosphere (Fe_2_O_3_-CMS) have been effectively produced by a hydrothermal method, and easily mixed with nitrocellulose to obtain NC-catalyst composites. The DSC analyses proved a high compatibility of the as prepared Fe_2_O_3_-CMS with a slight increase of the temperature of decomposition, suggesting the safety use of such NC-Fe_2_O_3_-CMS composite.
(2) The kinetic methods employed to determine the kinetics parameters revealed that the activation energy decreased by 12.9 kJ/mol with the presence of supported Fe_2_O_3_ nanoparticles, whereas the addition of CMS alone has no catalytic activity on the decomposition behavior of NC. Furthermore, the non-isothermal decomposition of nitrocellulose has been modeled. While TAS allowed the same kinetic reaction model (G_7_ = $[1-{\left(1-\alpha \right)}^{\frac{1}{2}}{]}^{1/2}$ for pure NC, the *it*-KAS and *it*-FWO models provided the same decomposition model D4 of three-dimensional diffusion (Ginstling–Brounshtein). It is demonstrated that NC-Fe*_2_*O_3_-CMS decomposes according to the nucleation power and parabolic low (P_2_ = $\alpha^{2}$, P_1/3_ = $\alpha^{\frac{1}{3}}$) for the three isoconversional models. The addition of carbon mesospheres, which does not significantly change the activation energy, affects the reaction model, where for *it*-KAS and *it*-FWO isoconversional models, NC-CMS decomposes according to the random nucleation mechanism (Avrami–Erofeev) A_2_ = $-\ln{\left(1-\alpha \right)}^{\frac{1}{2}}$, whereas a chemical reaction is obtained by the TAS model.
(3) The Fe_2_O_3_-CMS catalyst presents interesting features due to the stabilization effect before the decomposition point of energetic ingredient (NC), which is of great importance for the safety of energetic materials for long-term storage. Once the decomposition occurs, Fe_2_O_3_-CMS would accelerate the reactions and result in faster decomposition and higher energy releases.

## Figures and Tables

**Table 1:** The decomposition temperatures and heat release at various heating rates for the different samples.

Samples	*β* (°C/min)	*T*_onset_ (°C) ^(b)^	*T*_peak_ (°C) ^(a)^	∆*H* (J/g)
NC	5	193.3	202.3	−1214
	10	198.8	208.6	−1324
	15	202.9	213.7	−1427
	20	205.5	216.3	−1769
NC-CMS	5	192.4	202.5	−1284
	10	196.8	209.8	−1465
	15	201.4	213.8	−1479
	20	204.5	216.9	−1784
NC-Fe_2_O_3_-CMS	5	194.2	202.7	−1595
	10	196.8	209.8	−1900
	15	200.7	213.6	−2707
	20	203.6	216.9	−3092

^(a)^ Uncertainty *u* associated with the onset decomposition temperature is *u*(*T*_onset_) = ±0.4 K. ^(b)^ Uncertainty *u* associated with the top decomposition temperature is *u*(*T*_peak_) = ±0.2 K.

**Table 2:** The kinetic parameters of the investigated samples.

System	Isoconversional Method Kinetic Parameters				
			*E*_a_ (kJ/mol)	Log(*A*(S-1))	Reaction Model: g(α)
NC	it-KAS		170 ± 2	16 ± 3	$D_{4}=1-\left(\frac{2}{3}\right)\alpha -{\left(1-\alpha \right)}^{\frac{2}{3}}$
	*it*-FWO		170 ± 2	16 ± 3	$D_{4}=1-\left(\frac{2}{3}\right)\alpha -{\left(1-\alpha \right)}^{\frac{2}{3}}$
	TAS		170 ± 2	16 ± 2	$G_{7}=[1-(1-\alpha )2^{\frac{1}{2}}{]}^{1/2}$
	VYA/CE	*β* = 5 °C/min	170 ± 4	16 ± 5	
		*β* = 10 °C/min		16 ± 5	
		*β* = 15 °C/min		17 ± 5	
		*β* = 20 °C/min		17 ± 5	
NC-CMS	it-KAS		180 ± 1	17 ± 2	$A_{2}=-\ln{\left(1-\alpha \right)}^{\frac{1}{2}}$
	*it*-FWO		170 ±1	17 ± 1	$A_{2}=-\ln{\left(1-\alpha \right)}^{\frac{1}{2}}$
	TAS		180 ± 1	16 ± 1	$F_{3/4}=1-{\left(1-\alpha \right)}^{\frac{1}{4}}$, $R_{3}=F_{2/3}=1-{\left(1-\alpha \right)}^{\frac{1}{3}}$
	VYA/CE	*β* = 5 °C/min	180 ± 2	17 ± 3	
		*β* = 10 °C/min		17 ± 3	
		*β* = 15 °C/min		17 ± 3	
		*β* = 20 °C/min		17 ± 3	
NC-Fe_2_O_3_-CMS	it-KAS		160 ± 1	15 ± 1	$P_{2}=\alpha^{2}$
	*it*-FWO		160 ± 1	15 ± 1	$P_{2}=\alpha^{2}$
	TAS		160 ± 1	16 ± 1	$P_{1/3}=\alpha^{\frac{1}{3}}$ $,\;P_{1/4}=\alpha^{\frac{1}{4}}$
	VYA/CE	*β* = 5 °C/min	160 ± 2	15 ± 2	
		*β* = 10 °C/min		16 ± 2	
		*β* = 15 °C/min		16 ± 2	
		*β* = 20 °C/min		16 ± 2	

D_4_, Three-dimensional diffusion (Ginstling–Brounshtein); F_3/4_, Chemical reaction; A_2_, Random nucleation (Avrami–Erofeev); P_1/3_ and P_1/4_, nucleation (Power low); G_7_, Other kinetic equations with unjustified mechanism (TAS); P_2_, nucleation (parabolic low).

**Table 3:** Compensation parameters obtained by the Trache–Abdelaziz–Siwani (TAS) method and Vyazovkin’s equation (VYA/CE’s).

System	Log *A* = a *E*_a_ + b				
			a (mol/J)	b	R^2^
NC	TAS		0.2653 ± 5 × 10^−4^	−7.959 ± 0.088	0.99991
	VYA/CE	*β* = 5 °C/min	0.2518 ± 2 × 10^−5^	−6.225 ± 0.008	0.99936
		*β* = 10 °C/min	0.2518 ± 2 × 10^−5^	−5.532 ± 0.008	0.99936
		*β* = 15 °C/min	0.2518 ± 2 × 10^−5^	−5.126 ± 0.007	0.99936
		*β* = 20 °C/min	0.2518 ± 3 × 10^−5^	−4.839 ± 0.011	0.99936
NC-CMS	TAS		0.2202 ± 1 × 10^−5^	−1.164 ± 0.020	0.99816
	VYA/CE	*β* = 5 °C/min	0.2522 ± 3 × 10^−5^	−6.062 ± 0.013	0.999515
		*β* = 10 °C/min	0.2522 ± 2 × 10^−5^	−5.371 ± 0.006	0.999515
		*β* = 15 °C/min	0.2522 ± 3 × 10^−5^	−4.965 ± 0.013	0.999515
		*β* = 20 °C/min	0.2522 ± 3 × 10^−5^	−4.677 ± 0.011	0.999515
NC-Fe_2_O_3_-CMS	TAS		0.3024 ± 2 × 10^−5^	13.128 ± 0.026	0.99956
	VYA/CE	*β* = 5 °C/min	0.2528 ± 2 × 10^−5^	−6.073 ± 0.076	0.999541
		*β* = 10 °C/min	0.2528 ± 2 × 10^−5^	−5.380 ± 0.071	0.999541
		*β* = 15 °C/min	0.2528 ± 2 × 10^−5^	−4.975 ± 0.065	0.999541
		*β* = 20 °C/min	0.2528 ± 2 × 10^−5^	−4.687 ± 0.061	0.999541

## Supplementary Materials

The following are available online at https://www.mdpi.com/2079-4991/10/5/968/s1, Figure S1: SEM images treated with ImageJ (a, b) CMS, (c, d) CMS-Fe_2_O_3_; Figure S2: Particle size distribution using ImageJ software for MCS; Figure S3: Particle size distribution using ImageJ software for Fe_2_O_3_-MCS; Table S1: Statistics on columns of particle size distribution for MCS; Table S2: Statistics on columns of particle size distribution for Fe_2_O_3_-MCS.
