# Supplementary material for: Hydrothermal Synthesis of Hematite Nanoparticles Decorated on Carbon Mesospheres and Their Synergetic Action on the Thermal Decomposition of Nitrocellulose
Source: Nanomaterials (Basel). 2020 May 18;10(5):968. doi: 10.3390/nano10050968 (PMC7711595; doi:10.3390/nano10050968)
Supplement: Supplementary file 1 [file nanomaterials-10-00968-s001.pdf]

Supplementary Materials:

Hydrothermal Synthesis of Hematite Nanoparticles Decorated on Carbon Mesospheres and Their Synergetic Action on the Thermal Decomposition of Nitrocellulose

Abdenacer Benhammada [1,2], Djalal Trache [1,*], Mohamed Kesraoui [1] and Salim Chelouche [1]

[1] UER Procédés Energétiques, Ecole Militaire Polytechnique, BP 17, Bordj El-Bahri, Algiers 16046, Algeria; nbenhammada@yahoo.fr (A.B.); kesraoui.mohamed@gmail.com (M.K.); salim.chelouche@gmail.com (S.C.)

[2] Ecole Nationale Préparatoire Aux Etudes d'Ingénieur Badji Mokhtar, ENPEI, BP 5, Rouiba, Algiers 16013, Algeria

* Correspondence: djalaltrache@gmail.com

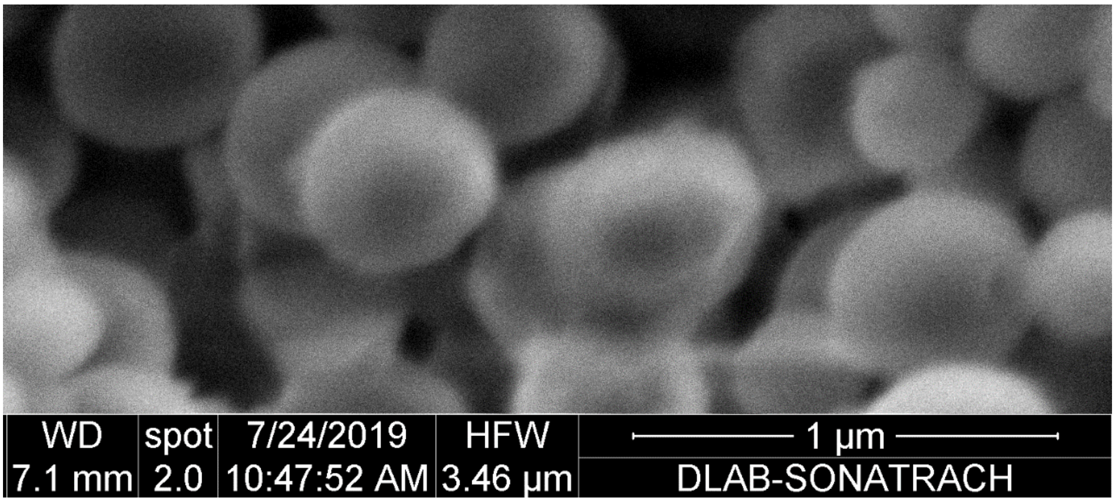

a

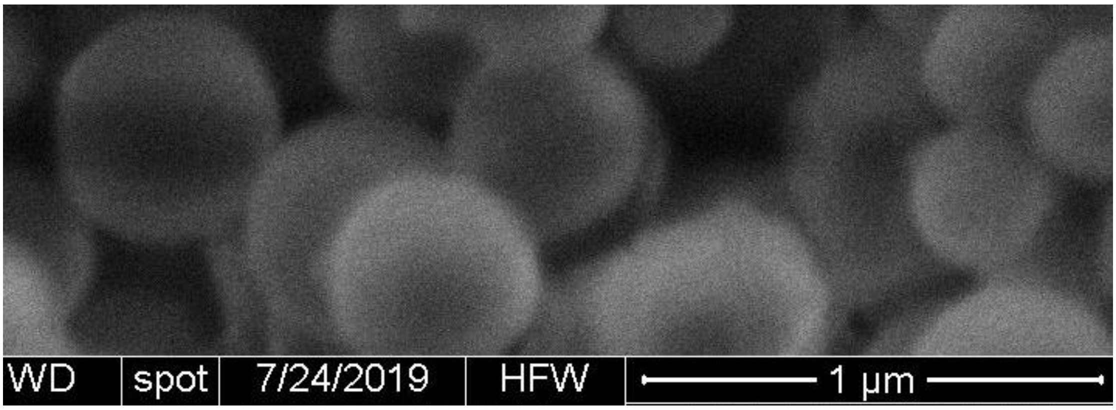

b

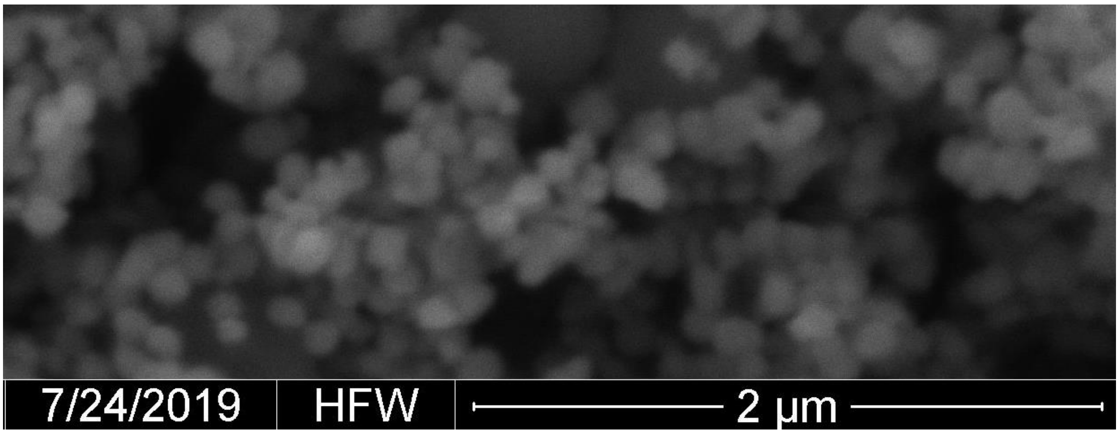

c

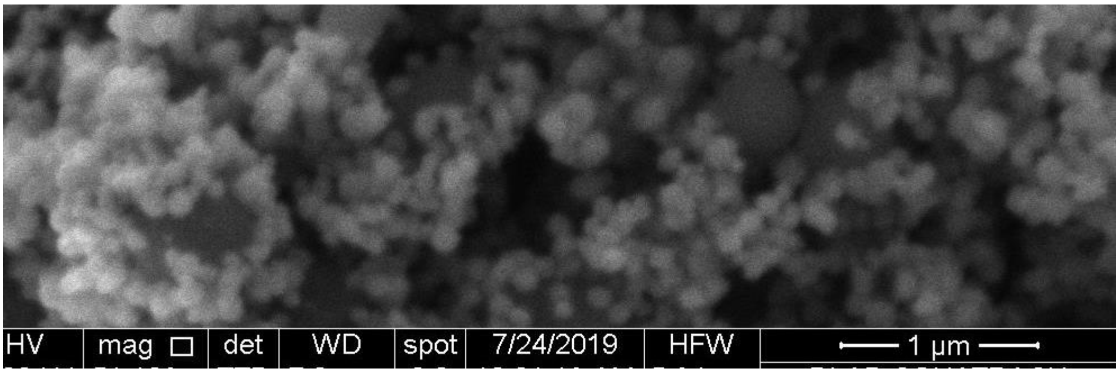

d

Figure S1. SEM images treated with ImageJ (**a**, **b**) CMS, (**c**, **d**) CMS-Fe_2O_3.

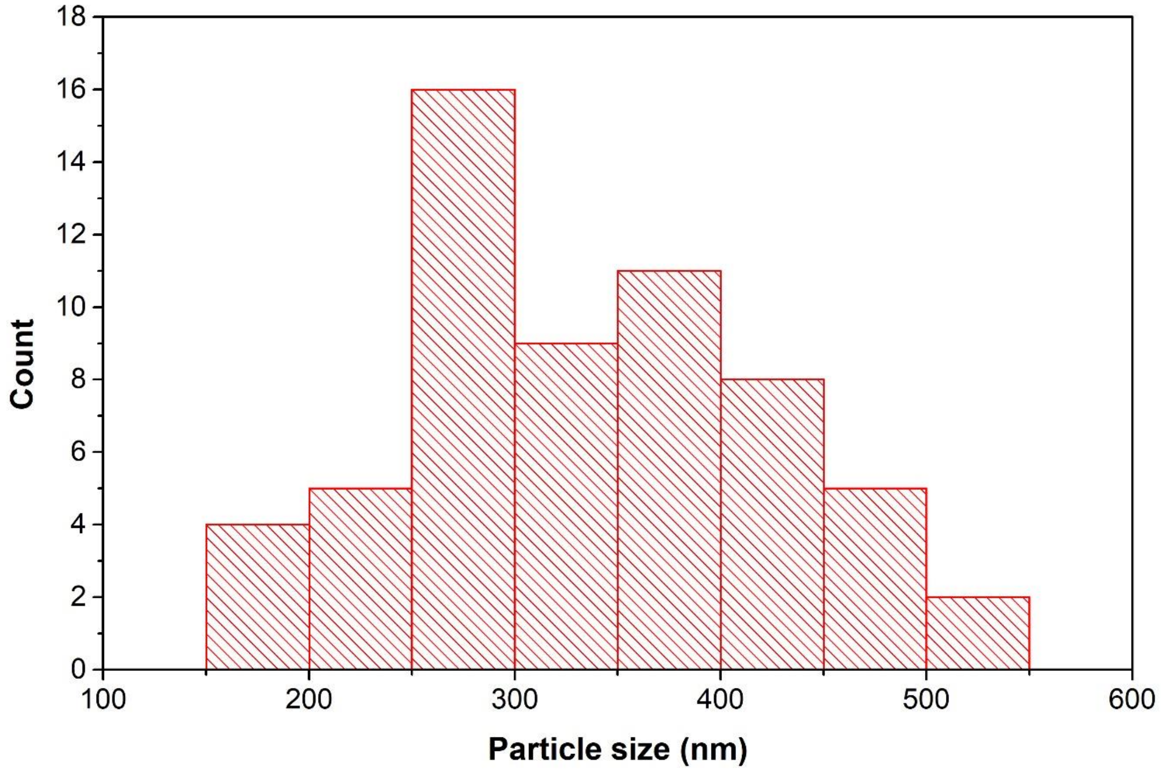

Figure S2. Particle size distribution using ImageJ software for MCS.

Table S1. Statistics on columns of particle size distribution for MCS.

**Total number of the treated particles**	**Mean (nm)**	**Standard Deviation**	**Sum**	**Minimum (nm)**	**Median (nm)**	**Maximum (nm)**
60	334.5	86.9	20071	156	319.5	513

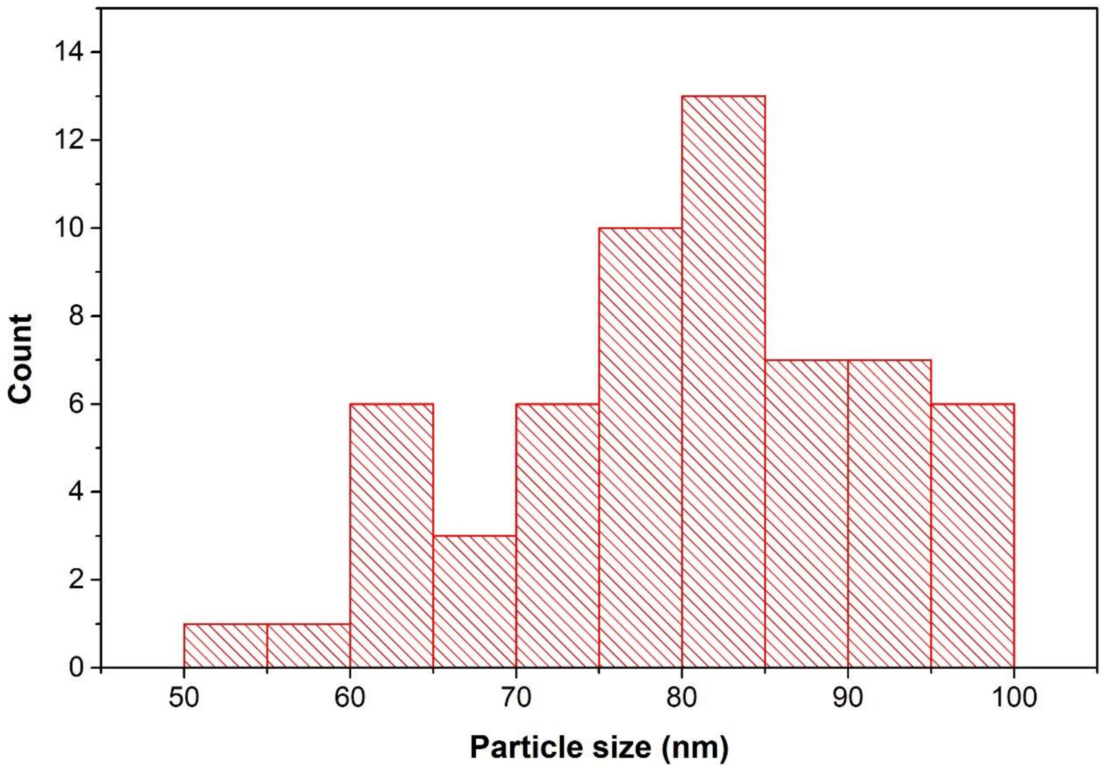

Figure S3. Particle size distribution using ImageJ software for Fe_2O_3-MCS.

Table S2. Statistics on columns of particle size distribution for Fe_2O_3-MCS.

Total number of the treated particles	Mean (nm)	Standard Deviation	Sum	Minimum (nm)	Median (nm)	Maximum (nm)
60	80.0	11.0	4801.8	54.5	81.1	98.0
